# Lack of Salt-Inducible Kinase 2 (SIK2) Prevents the Development of Cardiac Hypertrophy in Response to Chronic High-Salt Intake

**DOI:** 10.1371/journal.pone.0095771

**Published:** 2014-04-21

**Authors:** Sergej Popov, Hiroshi Takemori, Takeshi Tokudome, Yuanjie Mao, Kentaro Otani, Naoki Mochizuki, Nuno Pires, Maria João Pinho, Anders Franco-Cereceda, Lucia Torielli, Mara Ferrandi, Anders Hamsten, Patricio Soares-da-Silva, Per Eriksson, Alejandro M. Bertorello, Laura Brion

**Affiliations:** 1 Membrane Signaling Networks, Department of Medicine, Karolinska Institutet, CMM, Karolinska University Hospital-Solna, Stockholm, Sweden; 2 Laboratory of Cell Signaling and Metabolism, National Institute for Biomedical Innovation, Osaka, Japan; 3 Department of Biochemistry, National Cerebral and Cardiovascular Research Institute, Osaka, Japan; 4 Regenerative Medicine and Tissue Engineering, National Cerebral and Cardiovascular Research Institute, Osaka, Japan; 5 Cell Biology, National Cerebral and Cardiovascular Research Institute, Osaka, Japan; 6 BIAL - Portela & C^a^, S.A., S. Mamede do Coronado, Portugal; 7 MedInUP - Center for Drug Discovery and Innovative Medicines, University of Porto, Porto, Portugal; 8 Cardiothoracic Surgery Unit, Department of Molecular Medicine and Surgery, Karolinska Institutet, Stockholm, Sweden; 9 Prassis Sigma-Tau Research Institute, Settimo Milanese, Milan, Italy; 10 Cardiovascular Genetics and Genomics, Department of Medicine, Karolinska Institutet, CMM, Karolinska University Hospital-Solna, Stockholm, Sweden; Osaka University Graduate School of Medicine, Japan

## Abstract

Cardiac left ventricle hypertrophy (LVH) constitutes a major risk factor for heart failure. Although LVH is most commonly caused by chronic elevation in arterial blood pressure, reduction of blood pressure to normal levels does not always result in regression of LVH, suggesting that additional factors contribute to the development of this pathology. We tested whether genetic preconditions associated with the imbalance in sodium homeostasis could trigger the development of LVH without concomitant increases in blood pressure. The results showed that the presence of a hypertensive variant of α-adducin gene in Milan rats (before they become hypertensive) resulted in elevated expression of genes associated with LVH, and of salt-inducible kinase 2 (SIK2) in the left ventricle (LV). Moreover, the mRNA expression levels of SIK2, α-adducin, and several markers of cardiac hypertrophy were positively correlated in tissue biopsies obtained from human hearts. In addition, we found in cardiac myocytes that α-adducin regulates the expression of SIK2, which in turn mediates the effects of adducin on hypertrophy markers gene activation. Furthermore, evidence that SIK2 is critical for the development of LVH in response to chronic high salt diet (HS) was obtained in mice with ablation of the *sik2* gene. Increases in the expression of genes associated with LVH, as well as increases in LV wall thickness upon HS, occurred only in *sik2^+/+^* but not in *sik2^−/−^* mice. Thus LVH triggered by HS or the presence of a genetic variant of α-adducin requires SIK2 and is independent of elevated blood pressure. Inhibitors of SIK2 may constitute part of a novel therapeutic regimen aimed at prevention/regression of LVH.

## Introduction

Cardiac hypertrophy (CH) is a thickening of the heart muscle wall that reduces the volume of the cardiac chambers, and constitutes a major risk factor for heart failure [1], [2]. The most common form of CH (increased left ventricle mass) is caused by chronic high arterial blood pressure. However, even if blood pressure is normalized, CH may not regress completely. This suggests that additional factors, independently of high blood pressure, may contribute to the development of CH. Among those factors, genetics [3] (salt-sensitive individuals) and life style [4] (high salt content of the diet) are assumed to play a significant role.

High dietary salt content has been associated with increased left ventricle mass (LVM) in humans (regardless of the blood pressure) [5], [6] as well as in experimental animals [7]. Although several molecular targets have been proposed to be responsible for the development of CH [8], a critical molecular target within cardiac myocytes with an active role in this process (linking high salt intake to CH) has remained elusive. In rats, elevated salt intake triggers the expression of salt-inducible kinase (SIK) within the adrenal glands [9] and influences aldosterone synthesis [10]. SIK belongs to the AMPK family of kinases [11], [12] and consists of three isoforms. Involvement of SIK isoforms has been demonstrated in pathophysiological processes within various tissues, among them skeletal myocytes, where SIK1 promotes cell survival [13], and in the liver, where it regulates fast glucose metabolism [14]. Additionally, SIK1 participates in the regulation of cell sodium transport through association with, and activation of Na^+^,K^+^-ATPase upon increases in intracellular sodium [15] and in response to G protein-coupled receptors [16]. Blocking SIK1 prevents the renal cell hypertensive phenotype associated with the hypertensive variant of human α-adducin [17]. Recently, we observed that SIK1 also regulates Na^+^,K^+^-ATPase activity in vascular smooth muscle cells and is associated with variations in blood pressure and LVM [18]. SIK1 has been isolated from the myocardium [19], [20], and the lack of SIK1 in mouse embryonic stem cells impairs cardiomyogenesis [21].

Because SIK proteins are important cellular targets influencing normal myocardial development, sodium reabsorption and blood pressure, we hypothesized that genetic preconditions alone or in combination with elevated salt intake/abnormal sodium homeostasis could directly influence the development of CH via cardiac SIK networks and independently of high arterial blood pressure.

## Materials and Methods

### Ethics Statement

Human heart biopsies were taken from the left ventricle of patients undergoing heart valve surgery [22]. Subjects had given their written informed consent to participation. The study was approved by the ethics committee at the Karolinska Institutet, Stockholm, Sweden. All clinical investigation was conducted according to the principles expressed in the Declaration of Helsinki. Milan normotensive and hypertensive rats (30 days-old) were obtained from the Prassis Sigma-Tau Research Institute breeding unit. They were housed inside a limited access animal facility and had free access to food and water. The *sik2^−/−^* mouse (C57BL/6J background) has been previously described [23]. Male *sik2^−/−^* mice and wild-type litters were grouped (n = 7–10) and served Milli-Q water supplemented with or without 1% NaCl during the periods from 13 weeks old to 25 weeks old. All animals were maintained under standard conditions of light (7 a.m., 7 p.m.) and temperature (22°C, 55% humidity) and all surgical procedures were performed under anesthesia and efforts were made to minimize suffering. The care and husbandry of animals were in accordance with European Directives no. 86/609. Ethics committees at the Karolinska Institute, Prassis Research Institute, NIBIO, University of Porto, and NCVC approved all experimental protocols.

### Cell Line

Cell-based studies were performed using a cell line (HL-1) derived from mouse atrial myocytes [24]. This cell line retains the myocyte phenotype in culture. Cells were grown in Claycomb medium (Sigma) with addition of 10% fetal bovine serum, 100 units/ml penicillin and 100 µg/ml streptomycin, 1% L-glutamine, and 10 µM norepinephrine (Sigma). Cells were grown in flasks coated with fibronectin (25 µg fibronectin in 2 ml of 0.02% gelatin). Studies were performed when cells reached 70% confluence.

### Plasmids and Transfection

Plasmids for the expression of α-adducin (G460/S586: normotensive, W460/C586: hypertensive) were designed and constructed as described [25]. SIK2 wild-type (WT) and kinase-deficient (K49M) cDNAs are described elsewhere [23]. MEF2 and MEF2-mutant luciferase vectors were obtained from Zixu Mao (Departments of Pharmacology and Neurology, Emory University School of Medicine, Atlanta, GA, USA) [26]. BNP promoter tagged luciferase was obtained from Professor David Gardner, and β-MHC promoter tagged luciferase originated from Dr. Robert MacLellan. Transient transfections were performed using lipofection (LipofectAMINE 2000, Invitrogen) according to the manufacturers’ instructions. α-adducin siRNA (sc-43254), γ-adducin-siRNA (sc-29641) and sc-siRNA (sc-37007) were purchased from Santa Cruz Biotechnology. Transfection was performed according to the manufacturer’s recommendations.

### Determination of MEF2, BNP and β-MHC Promoter Activity

Cells were plated in 12-well plates and transiently transfected with either the normotensive or hypertensive variant of α-adducin. MEF2-, BNP-, β-MHC luciferase promoter construct and *Renilla* luciferase vector (50∶1 ratio), and either SIK2-WT, SIK2-K49M were used. After 36 h expression, each of the promoter-derived firefly luciferase and Renilla luciferase activities were analyzed with Dual Luciferase Kit (Promega, Madison, WI, USA) according to manufacturer’s protocol and the activity was expressed in arbitrary units, or percentage change of the ratio MEF2, BNP and β-MHC/Renilla luciferase.

### Expression of SIK Protein Isoforms in HL-1 Cells

Cell homogenates (total protein content 150 µg) were separated on SDS-PAGE. Proteins were transferred to PVDF membranes and Western Blot was performed using specific SIK2 and SIK1 antibody [27].

### Determination of SIK2 Activity

HL-1 cells that had been transformed with pEBG-SIK2 (expression vector for GST-fusion protein) [28] were lysed with 1 ml of IP lysis buffer (50 mM Tris-HCl (pH 8.0), 5 mM EDTA, 5 mM EGTA, 2 mM DTT, 50 mM glycerol 3-phosphate, 50 mM NaF, 1 mM NaVO4, 0.5% Triton X-100, 1 mM phenylmethylsulfonyl fluoride, 10 micro g/ml leupeptin, and 14 micro g/ml aprotinin). GST-SIK2 protein was purified with glutathione-Sepharose column (GE-Healthcare). To detect SIK2 and phospho-SIK2 (pT175), anti-SIK2 antibody and anti-phospho-SIK1 (pT182) that recognize the pT175 (phospho amino acid that reflects activation in SIK2 protein) were used [27]. All samples were run on the same SDS-PAGE and Western Blot was performed. The membranes were probed with an antibody against phosphorylated SIK1 and with a polyclonal antibody against SIK2 that recognize multiple epitopes in the protein (reflecting total amount of SIK2 on the blot). SIK2 activity was expressed as the ratio between the phosphorylated to the total amount of SIK2 expressed as previously described [18].

### Gene Expression Analysis

Human heart biopsies and heart samples from mice and rats were directly incubated with RNAlater (Ambion, Austin, TX) and homogenized with a FastPrep using Lysing Matrix D tubes (MP Biomedicals, Germany). Total RNA from tissue or HL-1 cells was isolated using Trizol (Invitrogen, Paisley, Scotland, UK) and RNeasy Mini kit (Qiagen, Hilden, Germany) including treatment with RNase-free DNase set (Qiagen) according to manufacturer’s instructions. Reverse transcription of total RNA (500 ng) was performed using RevertAid™ H Minus M-MuLV First Strand cDNA Synthesis Kit (Fermentas, Life Science). Rat, murine and HL-1 cDNA was amplified using StepOnePlus™ Real-Time PCR System (Applied Biosystems, Foster city, CA). The relative amount of the mRNA of interest was normalized against to RPLP0 - ribosomal protein, large, P0 - and TBP - TATA box binding protein - mRNAs using the comparative Ct-method. The cDNA from human heart biopsies (n = 139) was amplified as previously described [22].

### Immunohistochemistry

The tissue was fixed in formaldehyde and mounted in paraffin. Slides were stained with haematoxylin and eosin.

### Determination of Blood Pressure Levels

Systolic blood pressure was measured in Milan normotensive and hypertensive rats by tail pletysmography (BP recorder, U. Basile, Varese, Italy) in conscious rats. The reported values are the average of at least three different measurements. Telemetry was used for measuring blood pressure in *sik2^+/+^* and *sik2^−/−^* mice. The carotid artery implantation was adapted from previously published [29]. Mice were anesthetized by intraperitoneal injection (10 ml/kg) of ketamine (150 mg/kg), medetomidine (1 mg/kg) and butorfanol (1 mg/kg) in 0.9% saline. Briefly, animals were placed on their backs on a heated pad (37°C) and a 1–2 cm incision at the level of the ventral neck was performed. Salivary glands were gently separated and the right common carotid artery was carefully isolated. Telemetry transmitter was inserted into the carotid after cranial permanent ligature and temporary caudal occlusion. Catheter tip was positioned and secured in the aortic arch. The body of the device was slipped in a subcutaneous pocket and inserted through the same neck incision, on the left flank, as close as possible to the left hindlimb of the animal. Lastly, the skin was sutured with 6/0 skin suture. After recovery (5 days after surgery), telemetry probes were magnetically turned on and blood pressure, heart rate and activity were monitored for 6 days with Dataquest A.R.T. Acquisition software (DSI). Collected data was analyzed with Dataquest A.R.T. Analysis software (DSI).

### Cardiac Function and Morphology

The cardiac function and morphology were assessed using a Vevo 2100 device equipped with an MS-400 probe (VisualSonics Inc., Toronto, Canada). Mice anesthetized with isoflurane were laid in the supine position, and the parasternal left ventricular (LV) short-axis view was acquired. The thickness of the left ventricular anterior (LVAW) and posterior wall (LVPW) was measured by using M-mode echocardiograms. Fractional shortening (FS) was calculated as follows: FS (%) = {(LVDd-LVDs)/LVDd}×100; (LVDd, LV end-diastolic diameter; LVDs, LV end-systolic diameter).

### Hemodynamic Studies

After anesthesia with isoflurane, a 1F Mikro-Tip Ultra-Miniature Pressure-Volume catheter (Millar Instruments, Houston, TX) was inserted into the LV through the right carotid artery to record baseline hemodynamics in the closed chest with the Pressure-Volume Conductance System (Millar Instruments) connected to a physiological recorder (PowerLab system, AD Instruments, Mountain View, CA). All data were analyzed with the PVAN 3.4 software package from Millar Instruments.

### Statistics

Linear regression analysis was used to analyze the data obtained from human hearts biopsies. Quantitative data were statistically analyzed (mean, s.e.m., s.d., t-test or Mann-Whitney as appropriate) and plotted using GraphPad Prism software. P values less than 0.05 were considered statistically significant.

## Results

### Left Ventricle Hypertrophy Markers Increase without Elevated Arterial Blood Pressure

**Figure 1:**
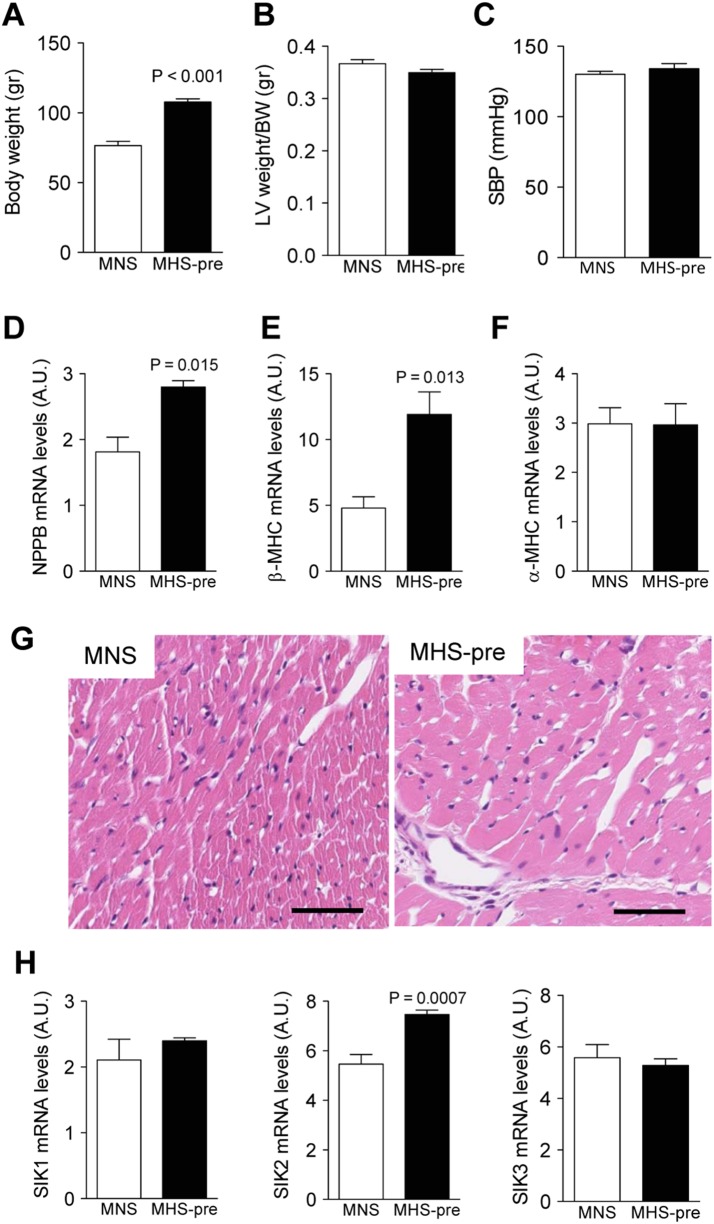
Expression of cellular markers of CH in normotensive- and pre-hypertensive Milan rats. Studies were performed in normotensive (MNS) and pre-hypertensive (MHS-pre) Milan rats at 30 days of age. Each bar represents the mean ± s.e.m., n = 6 animals in each group. **A)** Body weight; **B)** Left ventricular (LV) weight/body weight (BW) ratio; **C)** Systolic blood pressure (SBP); **D)** Brain natriuretic peptide (NPPB) mRNA expression levels; **E)** β-myosin heavy chain (β-MHC) mRNA expression levels; **F)** α-myosin heavy chain (α-MHC) mRNA expression levels; **G)** Representative hematoxylin and eosin stained cross-sections from the left ventricle MNS and MHS-pre rats. Bar: 50 µm; **H)** Expression levels of mRNA for SIK isoforms.

To examine whether development of the cell hypertrophic phenotype in cardiomyocytes may precede increases in arterial blood pressure, we used Milan hypertensive rats (MHS). This animal model harbors a genetic variation within the cytoskeletal protein α-adducin (*Add1* Phe316Tyr) [30], and all the rats that carry this variation will develop hypertension with age [31]. Thus, we could examine certain molecular markers associated with CH in hearts (left ventricle) from 30-day-old rats before they developed hypertension (MHS-pre) and compare to normotensive, 30-day-old matched controls (MNS). These rats, MHS-pre, have significantly higher body weight (Figure 1A), normal left ventricular weight indexed to body weight (Figure 1B), and, as expected, normal systolic blood pressure (Figure 1C) when compared to MNS. The mRNA levels of NPPB (gene for brain natriuretic peptide), α- and β-MHC (myosin heavy chain) were examined from left ventricle (LV) samples. The mRNA levels of NPPB (Figure 1D) and β-MHC (Figure 1E) were significantly increased in LV from MHS-pre, whereas α-MHC levels (Figure 1F) were similar to those in MNS. There were no obvious microscopic changes within the myocardium associated with hypertrophy between the MNS and MHS-pre hearts (Figure 1G), or in the expression levels of genes associated with cardiomyocyte remodeling/fibrosis (not shown). Interestingly, when we examined the mRNA expression levels for all three SIK isoforms (Figure 1H) we found that only SIK2 (*middle panel*), but not SIK1 (*left panel*) or SIK3 (*right panel*), was significantly elevated in hearts from MHS-pre when compared to MNS.

### SIK2 Mediates the Effect of Adducin on Hypertrophy Genes Expression

**Figure 2:**
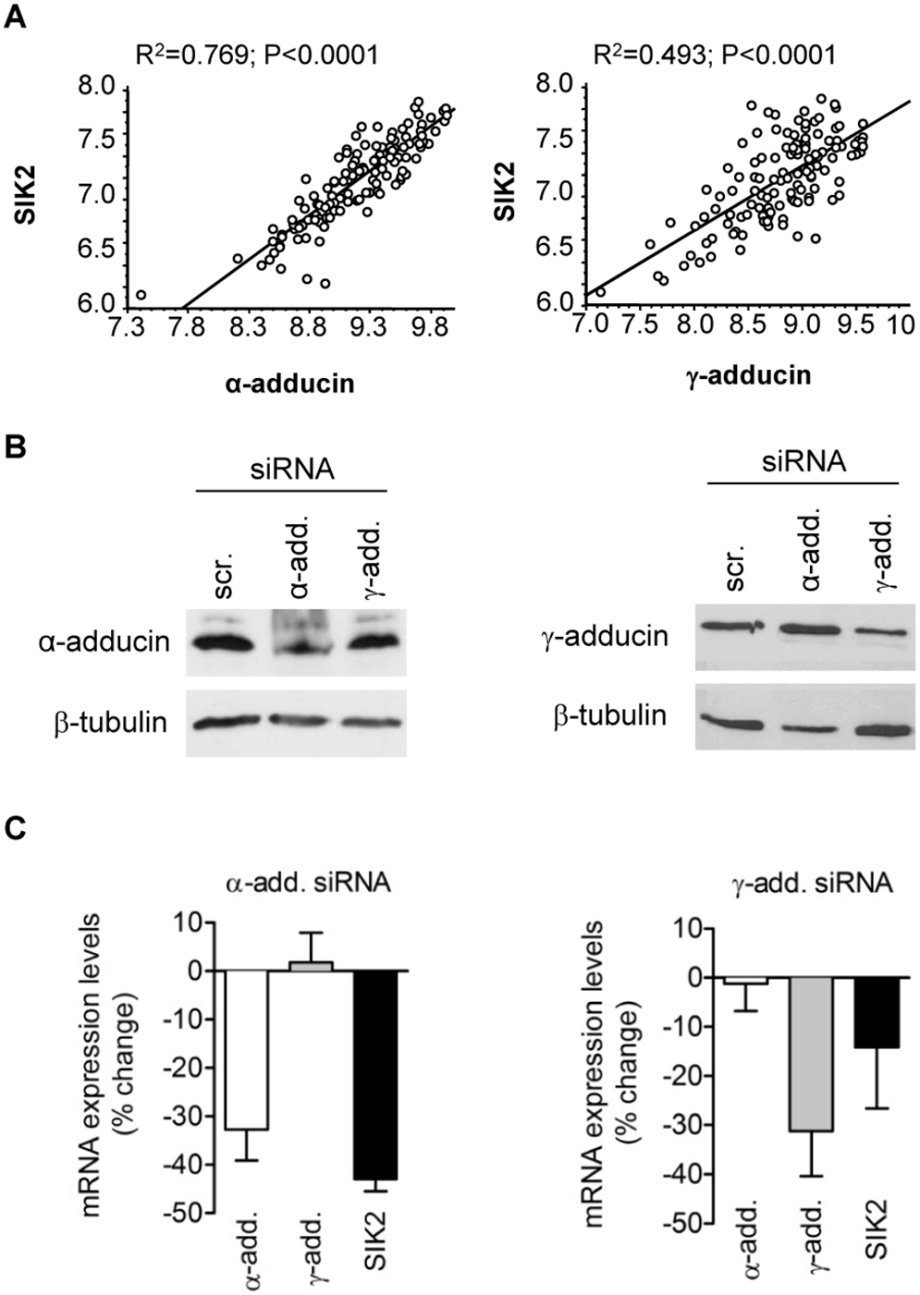
Adducin controls cellular SIK2 expression. A) Correlations between α-adducin (*left panel*) and γ-adducin (*right panel*) and SIK2 mRNA expression in biopsies obtained from human hearts. B) Representative Western blots of α- (*left panel*) and γ- adducin (*right panel*) in HL-1 cells transiently (48 h) transfected with α-add- (α-add.) or γ-add- siRNAs (γ-add.) and compared to control cells transfected with scrambled-siRNAs (scr.). Equal amount of protein (75 µg) was loaded in each condition. Antibodies used: α-adducin (1∶1000), γ-adducin (1∶1000) and loading control, β-tubulin (1∶1000). C) Expression levels (mRNA) of α-, γ- adducin, and SIK2 in cells transiently transfected with α-add- (*left panel*) or γ-add-siRNAs (*right panel*) vs. cells transfected with scrambled RNAs. Each bar represents the mean ± s.e.m., n = 4.

To examine whether adducin isoforms could influence the expression levels of SIK2, we first analyzed if there were correlations between the expression levels of α-, β-, and γ-adducin and the expression patterns of all three SIK isoforms at the mRNA level in samples obtained from tissue biopsies of 139 human hearts from patients undergoing heart valve surgery [22]. There was a positive and significant correlation between the expression levels of α- (Figure 2A, *left panel*) and γ-adducin (Figure 2A**,** *right panel*) and the expression of SIK2 but not between β-adducin and any SIK isoform (not shown). Furthermore, siRNA experiments in a cell line derived from mouse atrial myocytes (HL-1 cells) showed that the transient knockdown of α-adducin gene led to a decreased expression of SIK2 (Figure 2B and C, *left panel* *s*), whereas the knockdown of γ-adducin isoform did not significantly reduce SIK2 expression (Figure 2B and C, *right panel* *s*).

**Figure 3:**
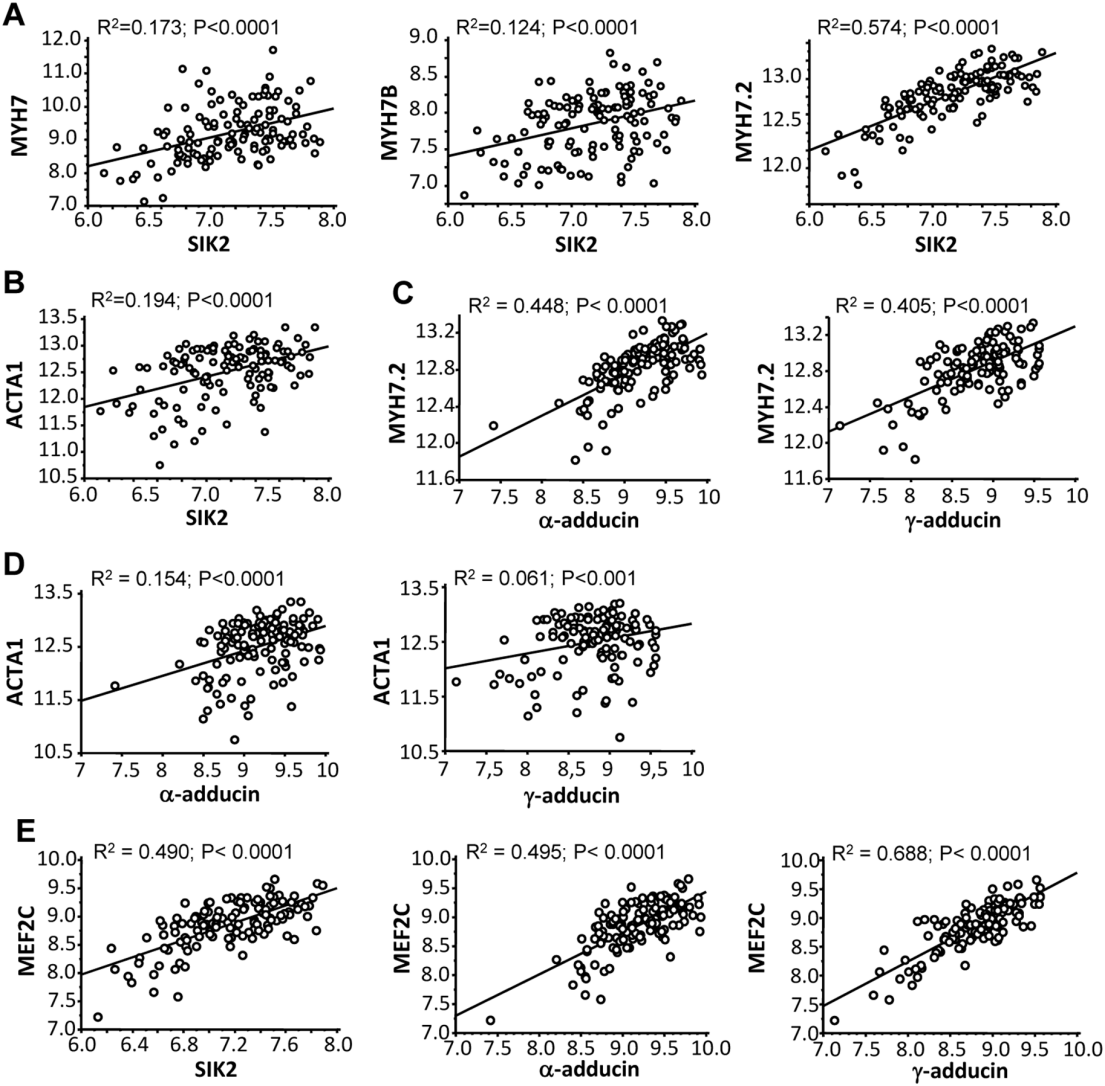
Correlations between mRNA levels of adducin and SIK2 with markers of CH in 139 biopsies from human hearts. A) Correlation between SIK2 and β-myosin heavy chain mRNA (MYH7) (*left panel*), cardiac specific β-myosin heavy chain mRNA (MYH7B) (*middle panel*) and sarcomere β-myosin heavy chain mRNA (MYH7.2) (*right panel*). B) Correlation between the mRNA expression levels of SIK2 and skeletal actin gene (ACTA1). C) Correlation between the mRNA expression levels of α-adducin (*left panel*) and γ-adducin (*right panel*) and sarcomere β-myosin heavy chain (MYH7.2). D) Correlation between the mRNA expression levels of α-adducin (*left panel*) and γ-adducin (*right panel*) and skeletal actin gene (ACTA1). E) Correlation between the mRNA expression levels of SIK2 (*left panel*), α-adducin (*middle panel*), γ-adducin (*right panel*) and MEF2C.

Next, the correlation between mRNA levels of SIK2, α- or γ-adducin with molecular markers of CH was further analyzed in the human hearts (Figure 3). The results showed a positive and significant correlation between the expression levels of SIK2 and β-MHC (Figure 3A**,** *left panel*), cardiac-specific β-MHC (Figure 3A **,** *middle panel*), sarcomere β-MHC (Figure 3A**,** *right panel*), and skeletal actin (ACTA1) (Figure 3B). Also, α- and γ-adducin correlated positively and significantly with sarcomere β-MHC (Figure 3C), and α- and γ-adducin with skeletal actin (Figure 3D). No correlation was found between β-adducin and any of the markers of CH (not shown). MEF2C (Myocyte-specific enhancer factor 2C, also known as MADS box transcription enhancer factor 2) represents an important transcription factor central to cardiomyocyte development, and it is relevant for the development of CH [32]. The mRNA expression levels of SIK2, α- and γ-adducin positively and significantly correlated with the expression patterns of MEF2C in these samples (Figure 3E).

**Figure 4:**
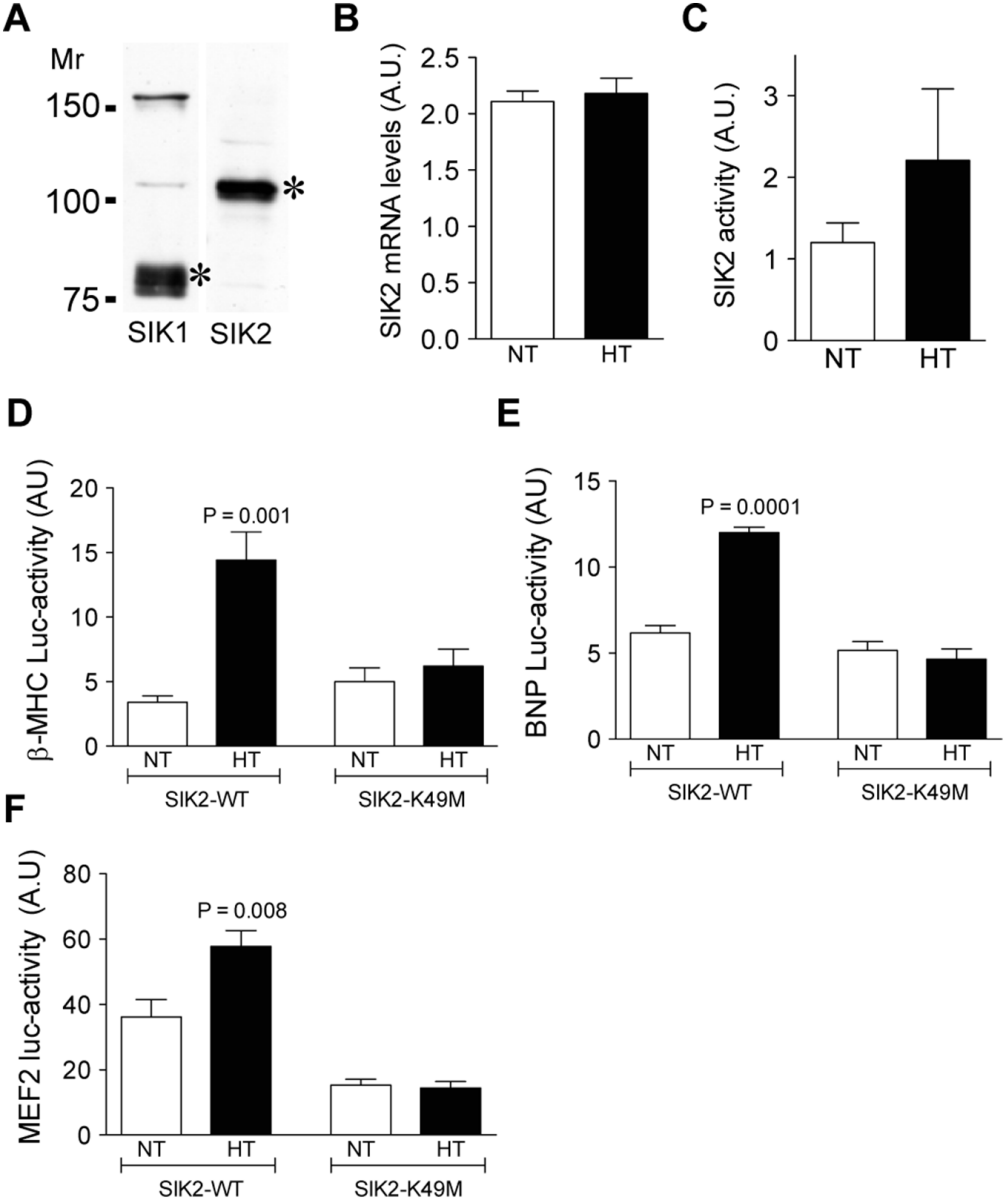
Acting via SIK2, adducin controls the expression of transcription factors and gene expression associated with CH. A) Presence of SIK1 and SIK2 isoforms in HL-1 cells in culture. An equal amount of protein (150 µg) was loaded in each condition. SDS-PAGE and Western blot was performed and a representative blot is shown. B) SIK2 mRNA expression levels in HL-1 cells expressing either the normotensive (NT) or hypertensive (HT) variant of α-adducin. C) SIK2 activity in HL-1 cells expressing either the normotensive (NT) or hypertensive (HT) variant of α-adducin. Bars represent the mean + s.e.m. of 6 experiments. D) β-myosin heavy chain (β-MHC) luciferase activity in HL-1 cells co-expressing either variant (NT or HT) of α-adducin with the SIK2 wild-type or a negative mutant (SIK2-K49M). E) Brain natriuretic peptide (BNP) luciferase activity in HL-1 cells expressing co-expressing either variant (NT or HT) of α-adducin with the SIK2 wild-type or a negative mutant (SIK2-K49M). F) MEF2C luciferase activity in HL-1 cells co-expressing either variant (NT or HT) of α-adducin with the SIK2 wild-type or a negative mutant (SIK2-K49M). Mean + s.e.m. of 6 experiments performed in duplicate are shown.

Given that the expression of α-adducin, SIK2 and the markers of CH are positively correlated in human hearts and that in MHS-pre hearts the presence of the hypertensive variant of α-adducin is associated with increased levels of SIK2 and CH markers, we speculate that the presence of α-adducin (hypertensive variant) could, by activating SIK2, sensitize to and trigger the molecular mechanism leading to CH. Adducin polymorphisms have been described also in humans [30], therefore we examined whether the presence of the human hypertensive variant of α-adducin (ADD1 Gly460Trp) was also associated with increased SIK2 expression, as observed in MHS-pre rats. Experiments were performed in HL-1 cells transiently overexpressing either the normotensive or the hypertensive variant of α-adducin. This cell line expresses the SIK2 isoform (Figure 4A). Transient overexpression of the hypertensive α-adducin did not change the SIK2 mRNA expression (Figure 4B), but it did result in an increase (a trend that did not reach statistical significance) in SIK2 catalytic activity (Figure 4C) when compared to cells transiently overexpressing the normotensive variant. Finally, we examined whether SIK2 could mediate the effects of the hypertensive variant of α-adducin on the expression levels of markers of CH (β-MHC and NPPB), as inferred from the observations in MHS-pre rats. The effects of α-adducin on β-MHC and NPPB expression were studied in HL-1 cells overexpressing one of the two α-adducin variants (normotensive or hypertensive) together with either the SIK2 wild-type or the SIK2 dominant negative mutant (K49M, which lacks kinase activity) [28]. The results clearly demonstrated that the transient overexpression of the hypertensive variant of α-adducin significantly increased β-MHC (Figure 4D) and NPPB (Figure 4E) expression, effect that was blocked in cells overexpressing the SIK2 defective kinase. The latter observation confirms the hypothesis that SIK2 at least partly mediates the effects of adducin on CH gene expression. Furthermore, the presence of the hypertensive variant of α-adducin led to increased MEF2C activity, and this effect was absent in cells overexpressing the SIK2 K49M mutant (Figure 4F).

### SIK2 is Critical for the Development of Left Ventricle Hypertrophy in Response to Chronic High-salt Intake

**Figure 5:**
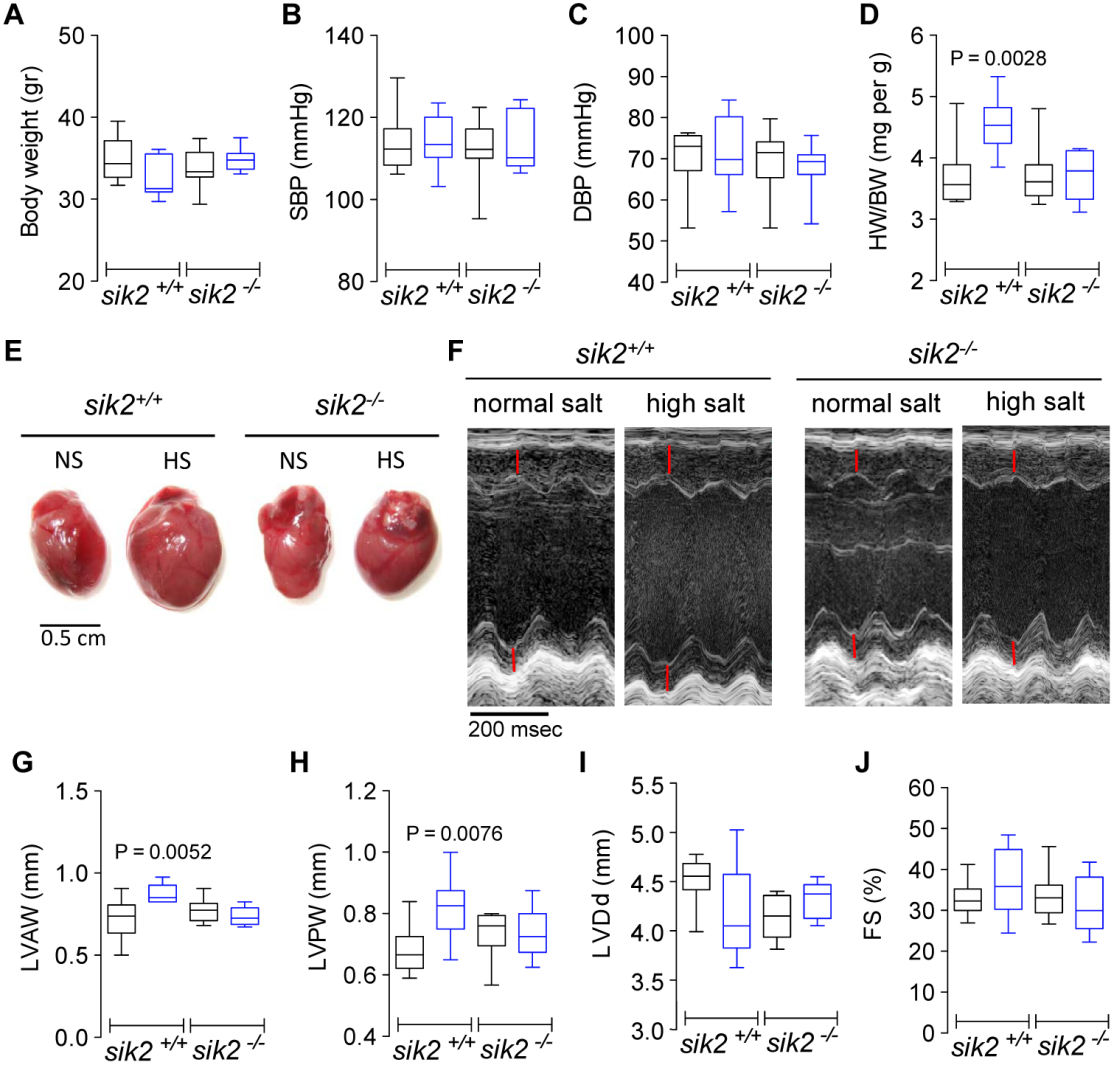
Effects of high salt diet on blood pressure and left ventricle size in *sik2^+/+^* and *sik2^−/−^* mice. Black bars correspond to mice on normal salt diet, whereas blue bars correspond to mice on high salt diet. A) Body weight, B) Systolic blood pressure (SBP), C) Diastolic blood pressure (DBP) and D) Heart weight/body weight (HW/BW) ratio. E) Representative gross images of whole hearts, and F) Representative ultrasound scans. Quantitative analysis of G) left ventricular anterior wall (LVAW) thickness, H) left ventricular posterior wall (LVPW) thickness, I) Left ventricular end-diastolic diameter (LVDd) and J) Fractional shortening (FS). Mean ± s.e.m. of 7–9 mice in each group.

MHS rats at 30 days of age have not yet developed hypertension, but their body weight, glomerular filtration rate and renal tubule Na^+^,K^+^-ATPase and Na^+^/K^+^/Cl^−^ co-transporter activity are all increased. Therefore, we speculated that SIK2 may not only mediate the effects of genetic variants of α-adducin on CH, but also represents a sensor for the sodium imbalances that result in abnormal cardiac growth. To test this hypothesis, we used a transgenic mouse with an ablation of the *sik2* gene [23]. Transgenic animals (*sik2^−/−^*) and their wild-type counterparts (*sik2^+/+^*) aged 4 months were given either water alone (normal salt) or with 1% saline (high salt) as drinking fluid. After 14 weeks, *sik2^+/+^* and *sik2^−/−^* mice drinking normal or high salt water maintained plasma electrolytes homeostasis (Table 1) and had similar and normal body weight (Figure 5A), systolic (SBP) (Figure 5B), and diastolic blood pressure (DBP) (Figure 5C), as well as heart rate (b/min: *sik2^+/+^*-NS: 479±50 vs. *sik2^+/+^*-HS: 493±43, and in *sik2^−/−^*NS: 488±44 vs. *sik2^−/−^*HS: 472±29). Among the mice receiving high salt water, the heart weight increased significantly in the *sik2^+/+^*, but it remained unchanged in *sik2^−/−^* mice (Figure 5D). Gross images of the whole heart demonstrated that high salt intake was associated with larger hearts in *sik2^+/+^* mice when compared to *sik2^−/−^* mice and to those given normal salt (Figure 5E). The increased size of the LV walls was also confirmed by echocardiography in living animals (Figure 5F). Whereas high salt intake significantly increased the LV anterior wall (Figure 5G) and posterior wall thickness (Figure 5H) in *sik2^+/+^* mice, this effect was absent in *sik2^−/−^* mice. The LV end diastolic diameter as well as the fractional shortening (Figure 5I and J) was not significantly different between the different genotypes, or between mice with different salt content of the diet. The hemodynamic data (Table 2) indicate that the LVH induced by long-term high salt intake in *sik2^+/+^* mice was associated with enlargement of LV end- systolic and end- diastolic volumes, and reduction of ejection fraction, although without significant change of cardiac output, dP/dt max and dP/dt min. In *sik2^−/−^* mice, the LV volumes were not significantly changed after the administration of a high salt diet, whereas the stroke volume and ejection fraction were increased. These data suggest that apart from a subtle increase of preload in *sik2^−/−^* as well as *sik2^+/+^* mice, there exists an obviously advanced heart remodeling in *sik2^+/+^* mice in response to chronic high salt intake.

**Table 1:** Serum electrolytes.

	mice	normal salt	high salt	P-values
Sodium, mE/L	*sik2^+/+^*	148±2.0	147±2.1	n.s.
	*sik2^−/−^*	149±1.3	148±1.0	n.s.
Potassium, mE/L	*sik2^+/+^*	5.7±1.1	6.4±0.8	n.s.
	*sik2^−/−^*	5.6±1.8	5.2±1.1*	n.s.
Chloride, mE/L	*sik2^+/+^*	113±1.7	115±2.2	n.s.
	*sik2^−/−^*	112±1.6	114±2.0	n.s.

mE/L =  milliequivalents per liter. Values are means ± SD. *p = 0.038 (*sik2^+/+^* vs. *sik2^−/−^* under high salt).

**Table 2:** Hemodynamic studies.

	mice	normal salt	high salt	P-values
LVEDV, µl	sik2^+/+^	31.4±4.6	36.9±4.5	0.0383
	sik2^−/−^	36.6±5.7	34.9±6.5	n.s.
LVESV, µl	sik2^+/+^	11.7±2.6	19.6±3.3	0.0002
	sik2^−/−^	14.5±3.6	11.6±4.7	n.s.
LVEDP, mmHg	sik2^+/+^	6.78±2.9	11.67±2.8	0.0065
	sik2^−/−^	7.64±3.1	7.51±2.6	n.s.
LVESP, mmHg	sik2^+/+^	100.2±5.2	107.9±11.8	n.s.
	sik2^−/−^	94.7±7.8	99.9±6.7	n.s.
EF %	sik2^+/+^	67.5±6.9	57.0±2.1	0.0034
	sik2^−/−^	63.1±7.8	73.3±8.1	0.0153
Stroke Volume, µl	sik2^+/+^	22.7±3.9	23.2±2.2	n.s.
	sik2^−/−^	23.3±4.8	27.3±4.1	0.0191
Cardiac output, µl/min	sik2^+/+^	10341±3673	11505±1586	n.s.
	sik2^−/−^	11521±3286	12937±2188	n.s.
dP/dt max, mmHg/sec	sik2^+/+^	8352±2149	7657±1091	n.s.
	sik2^−/−^	7994±2147*	8880±2645***	n.s.
dP/dt min, mmHg/sec	sik2^+/+^	−7886±867	−7989±1150	n.s.
	sik2^−/−^	−7487±1704	−7832±1166	n.s.

LVEDV  =  left ventricular end-diastolic volume, LVESV  =  left ventricular end-systolic volume, LVEDP  =  left ventricular end-diastolic pressure, LVESP  =  left ventricular end-systolic pressure, EF  =  ejection fraction. Values are means ± SD.

*p = 0.05 (*sik2^+/+^* vs. *sik2^−/−^* under normal salt),

***p = 0.001 (*sik2^+/+^* vs. *sik2^−/−^* under high salt).

At the molecular level, the mRNA expression of markers of cardiac hypertrophy was significantly higher in the LV from *sik2^+/+^* mice drinking 1% saline vs. normal salt (β-MHC = A.U.: 0.53±0.05 vs. 1.23±0.26, *p = 0.025*; MEF2C = A.U.: 0.38±0.02 vs. 0.62±0.01, *p = 0.045*), but not in *sik2^−/−^* mice.

Together, these data indicate that LVH triggered by chronic high salt intake is mediated by SIK2 action independently of blood pressure levels.

## Discussion

The results from this work demonstrate that SIK2 is responsible for the cellular/molecular events in cardiac myocytes that lead to LVH. Furthermore, the mediatory effect of SIK2 becomes relevant during high salt intake/plasma volume expansion, and in animals carrying a genetic variant of the cytoskeletal protein α-adducin, which is also associated with imbalance in sodium homeostasis. More importantly, these results indicate that high salt intake - independently of altered (elevated) arterial blood pressure - may result in CH via SIK2 action. The strength of these observations rests on the fact that we have used human heart material to establish correlation between the expression levels of SIK2 and those of established markers of CH, and subsequently performed *in vitro* and *in vivo* studies that confirmed their interdependence.

SIK isoforms (in particular SIK1) have been shown to act as mediators during the cellular adaptation to changes in intracellular sodium concentration. Although ubiquitous, this effect is particularly relevant in the renal epithelia [15], and in cardiac myocytes [33]. In cardiac myocytes, SIK1 mediates the increases in MEF2C, NFAT5c and genes associated with CH in response to increases in intracellular sodium [33]. However, the present study demonstrates that *in vivo* the SIK2 isoform, rather than SIK1, is more relevant for abnormal LV growth. Experiments using *sik2^−/−^ and sik2^+/+^* mice showed a direct link between the presence of SIK2 and the development of CH triggered by chronic high salt intake. Whereas this study demonstrates its importance in cardiac myocytes, it does not exclude the possibility that SIK2 present in other cell types could also be of relevance by affecting the release and/or the action of unknown cellular mediators activated in response to the higher content of salt in the diet. Ideally, the relative contribution of myocardial vs. non-myocardial SIK2 should be studied in animal models selectively lacking the cardiac SIK2 isoform. However, even without this information, it appears that blocking SIK2 activity/expression may be an important way to prevent the increase in LVM occurring in response to a high salt intake and/or to abnormal elevations in arterial blood pressure.

In Milan pre-hypertensive rats, the presence of the hypertensive variant of α-adducin was associated with elevated mRNA levels of markers of LVH and SIK2. The occurrence of these changes in the absence of other significant cardiovascular events indicates that this is an early step during LVH development and suggests that the presence of the hypertensive variant of α-adducin constitutes *per se* a risk factor for LVH. The lack of fibrosis and of increase in heart size observed in young MHS-pre rats is understandable because these are signs of an advanced cardiac hypertrophic phenotype that becomes evident in adult Milan hypertensive rats.

The observations obtained from Milan rats were also supported by correlation studies performed in myocytes from human hearts. Only α- and γ-adducin (but not β-adducin) positively correlate with the mRNA levels of SIK2 and molecular markers of LVH, and the same correlation was observed between SIK2 and these molecular markers. Even though the correlation studies *per se* do not provide a strong support for the importance of adducins and SIK2, they do add evidence to support the overall hypothesis that α-, γ-adducin and SIK2 are involved in the development of LVH, when taken together with the results from rat, mouse and *in vitro* studies. Importantly, specific knockdown of adducin isoforms using siRNAs in mouse cardiac myocytes showed that α-adducin in particular controls SIK2 mRNA expression. The molecular mechanism for this phenomenon remains to be explored. Furthermore, the increase of β-MHC and NPPB expression in cardiac myocytes overexpressing the hypertensive variant of α-adducin required SIK2 activity. Whereas in Milan pre-hypertensive rats the increased levels of markers of LVH are mediated solely by the presence of the hypertensive α-adducin variant acting via SIK2 alone or in combination with other factors that are influenced by altered sodium homeostasis remains unknown. At this young age, these animals do not show high blood pressure, but they may have renal dysfunction (increase in glomerular filtration rate [34] and increase in Na^+^,K^+^-ATPase activity along several nephron segments [31]) leading to sodium retention, and possibly to an increase in plasma volume reflected by the increased body weight.

How is the effect of a high salt diet translated into LVH without increases in arterial blood pressure? In this study we have not attempted to address this question. However, we can speculate that the effect could be achieved directly by a higher (absolute) content of sodium in the extravascular space (exchangeable sodium) due to its high content in the diet [35] and without necessarily reaching a higher plasma concentration (mE/L). Also, CH could be mediated through humoral factors such as endogenous ouabain (EO) or still unknown mediators released in response to high salt diet. Indeed, EO levels increase upon a high salt intake [36] and it has been demonstrated that elevated levels of circulating EO are associated with LV remodeling and greater LV mass in hypertensive rats [37]. Although this possibility is attractive, EO has generally been associated with elevated arterial blood pressure, and we did not observed such elevation in blood pressure in the Milan pre-hypertensive rats (at the time where studies were performed) nor in the *sik2^+/+^* mice.

Several studies in the past have demonstrated the relevance of diverse signaling molecules for normal/abnormal development of the heart. However, with the exception of few studies [38], [39], [40], [41], direct evidence for the involvement of these signaling molecules in LVH is scarce. In this study, we identify SIK2 as a molecule of relevance for the development of pathological hypertrophy due to high salt intake.

Our results open the possibility for a new therapeutic regimen targeting SIK2 during CH. Furthermore, agents that block SIK2 may not only be of benefit by preventing the development of CH in patients at risk (salt-sensitive), but also by promoting the regression of already established CH.
